# Supplementary material for: Redundant and non-redundant cytokine-activated enhancers control Csn1s2b expression in the lactating mouse mammary gland
Source: Nat Commun. 2021 Apr 14;12:2239. doi: 10.1038/s41467-021-22500-w (PMC8047016; doi:10.1038/s41467-021-22500-w)
Supplement: Supplementary file 1 — Supplementary Information [file 41467_2021_22500_MOESM1_ESM.pdf]

Supplementary Information

Redundant and non-redundant cytokine-activated enhancers control *Csn1s2b* expression in mouse mammary gland during lactation

Hye Kyung Lee[1, *], Michaela Willi[1], Tyler Kuhns[1], Chengyu Liu[2] and Lothar Hennighausen[1, *]

[1]Laboratory of Genetics and Physiology, National Institute of Diabetes and Digestive and Kidney Diseases, US National Institutes of Health, Bethesda, Maryland 20892, USA.

[2]Transgenic Core, National Heart, Lung, and Blood Institute, US National Institutes of Health, Bethesda, Maryland 20892, USA.

*Correspondence to: H.K.L (hyekyung.lee@nih.gov) and L.H (lotharh@niddk.nih.gov)

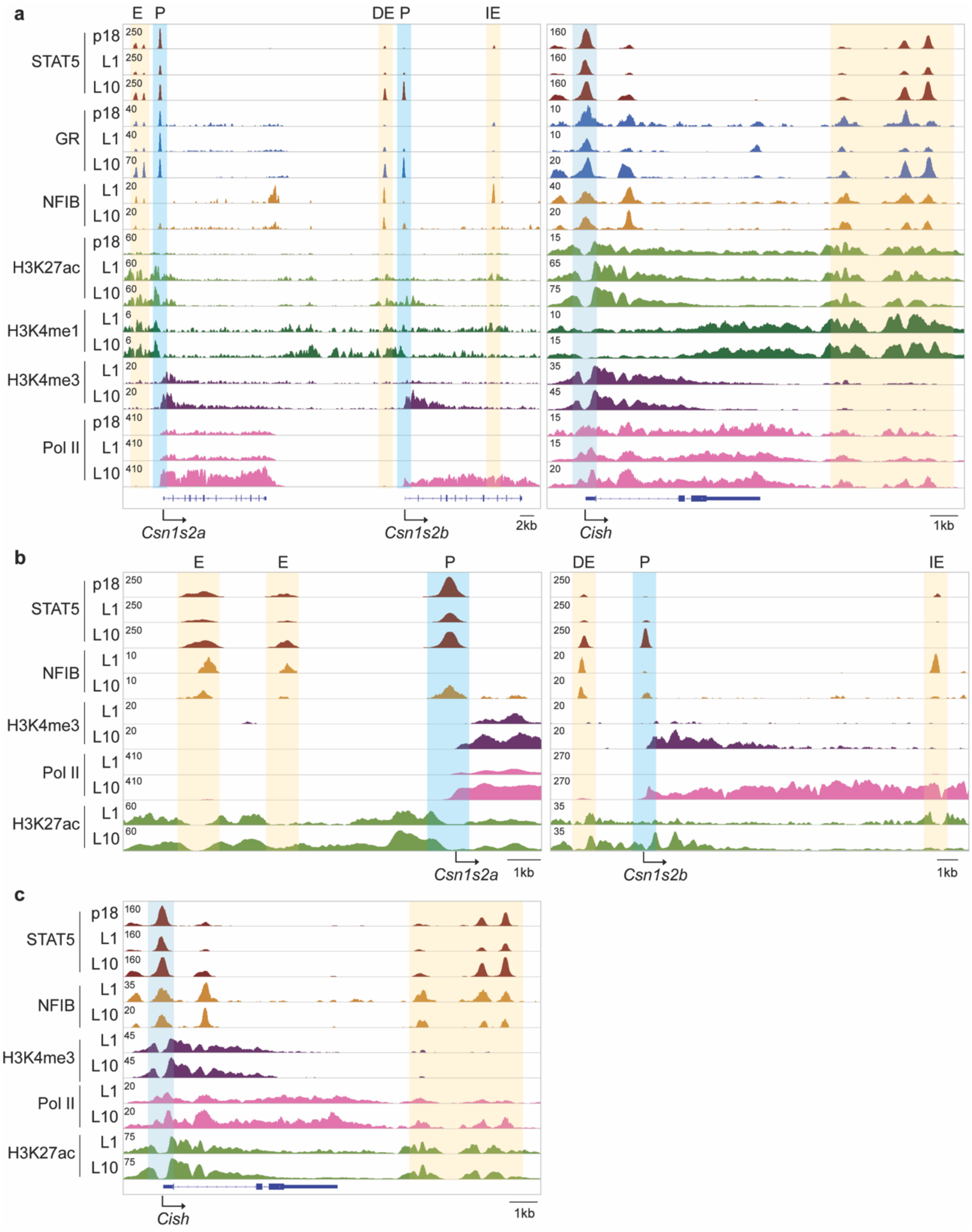

Supplementary Fig. 1 Genomic features of the *Csn1s2a/b* locus in mammary tissue during pregnancy and lactation. **a-b** Characteristics of the *Csn1s2a* and *Csn1s2b* loci, including NFIB binding and H3K27ac marks. DE, distal enhancer; P/E, promoter or enhancer; IE, intronic enhancer. **c** The *Cish* locus served as a ChIP-seq control for WT tissue at day 18 of pregnancy (p18) and day 1 of lactation (L1) and L10.

Rank	*de novo* Motif	p-value	% of Targets	Best match
1	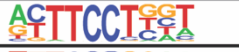	1e-2936	62.10%	ELF3(ETS)
2	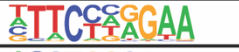	1e-2807	27.45%	STAT5(Stat)
3	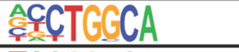	1e-1037	47.85%	HIC1(Zf)
4	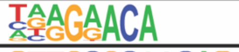	1e-748	57.98%	NR-halfsite
5	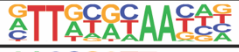	1e-578	5.85%	NFIL3(bZIP)
6	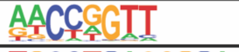	1e-272	15.17%	TFCP2
7	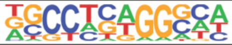	1e-228	14.76%	AP-2gamma(AP2)
8	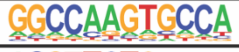	1e-192	8.82%	NFIX
9	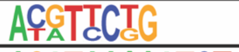	1e-145	12.99%	SPDEF(ETS)
10	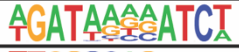	1e-125	1.66%	GATA(Zf)
11	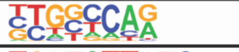	1e-109	17.55%	Bcl11a(Zf)
12	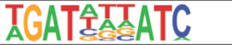	1e-107	2.57%	GATA(Zf)
13	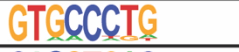	1e-66	9.16%	PB01331.1_Hic1_2
14	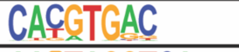	1e-61	7.6%	TEFC
15	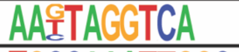	1e-52	0.57%	RORg(NR)
16	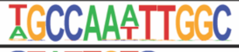	1e-52	0.22%	NFIA
17	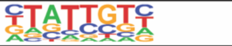	1e-36	2.55%	PB0183.1_Sry_2
18	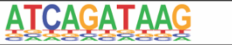	1e-17	0.14%	Gata1(Zf)

Supplementary Fig. 2 *De novo* Motif analysis for glucocorticoid receptor (GR) binding sites in mammary tissue at day ten of lactation (L10). Approximately 26,000 sites bound by GR were identified and 22,675 coincided with H3K27ac, indicative of candidate regulatory elements. Motifs for transcription factors (ETS factors, STAT5 and Nuclear Factor I family) know to be important for mammary development and function were significantly enriched at the 22,617 sites bound by GR and marked by by H3K27ac (±500 bp). No overt GR binding motifs were detected. Of 22617 of total target sequences and 26450 of total background sequences, Poisson test over *de novo* motif region were used to calculate p-value.

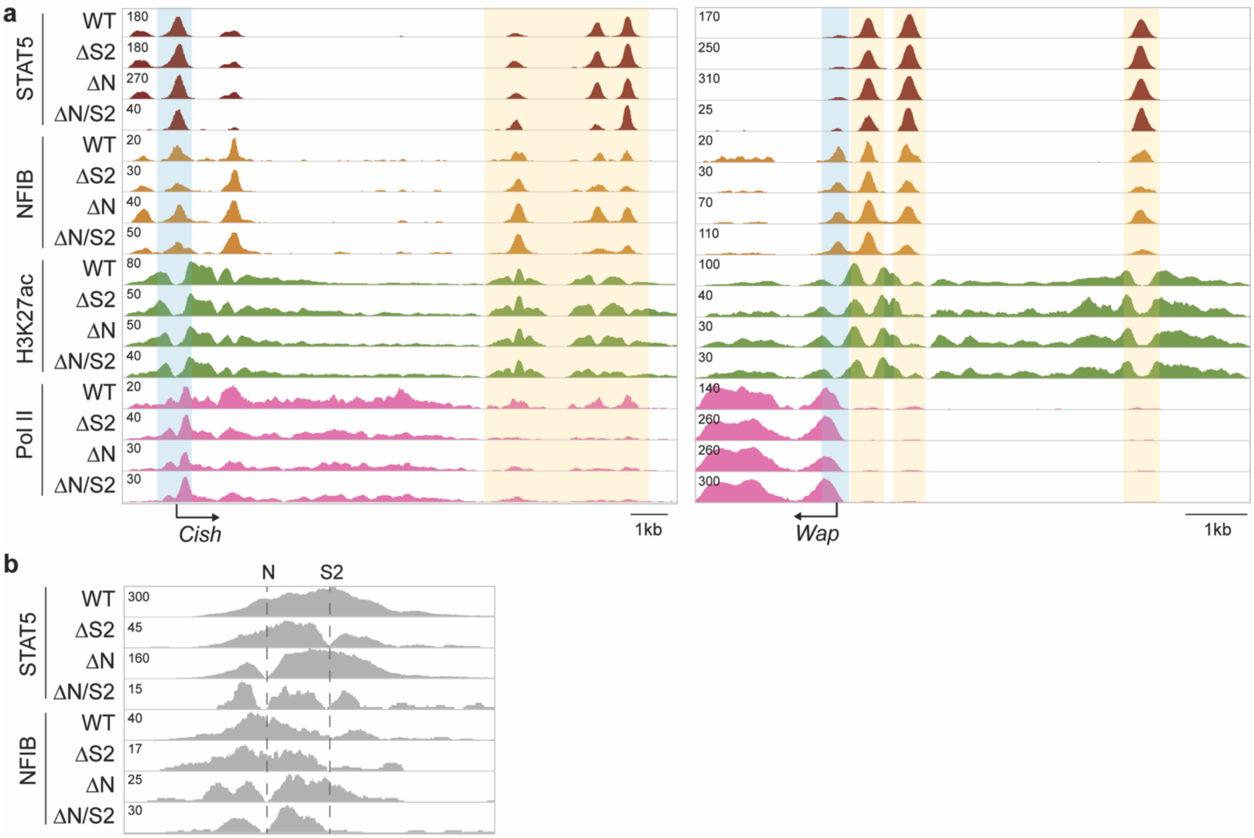

Supplementary Fig. 3 Limited impact of NFIB and STAT5 motif deletion in the *Csn1s2b* enhancer establishment. **a** The *Cish* and *Wap* served as ChIP-seq control for WT and mutant tissues at L10. **b** The raw mapping data of ChIP-seq confirmed the TF deletion and displays the TF binding activity in the *Csn1s2b* enhancer.

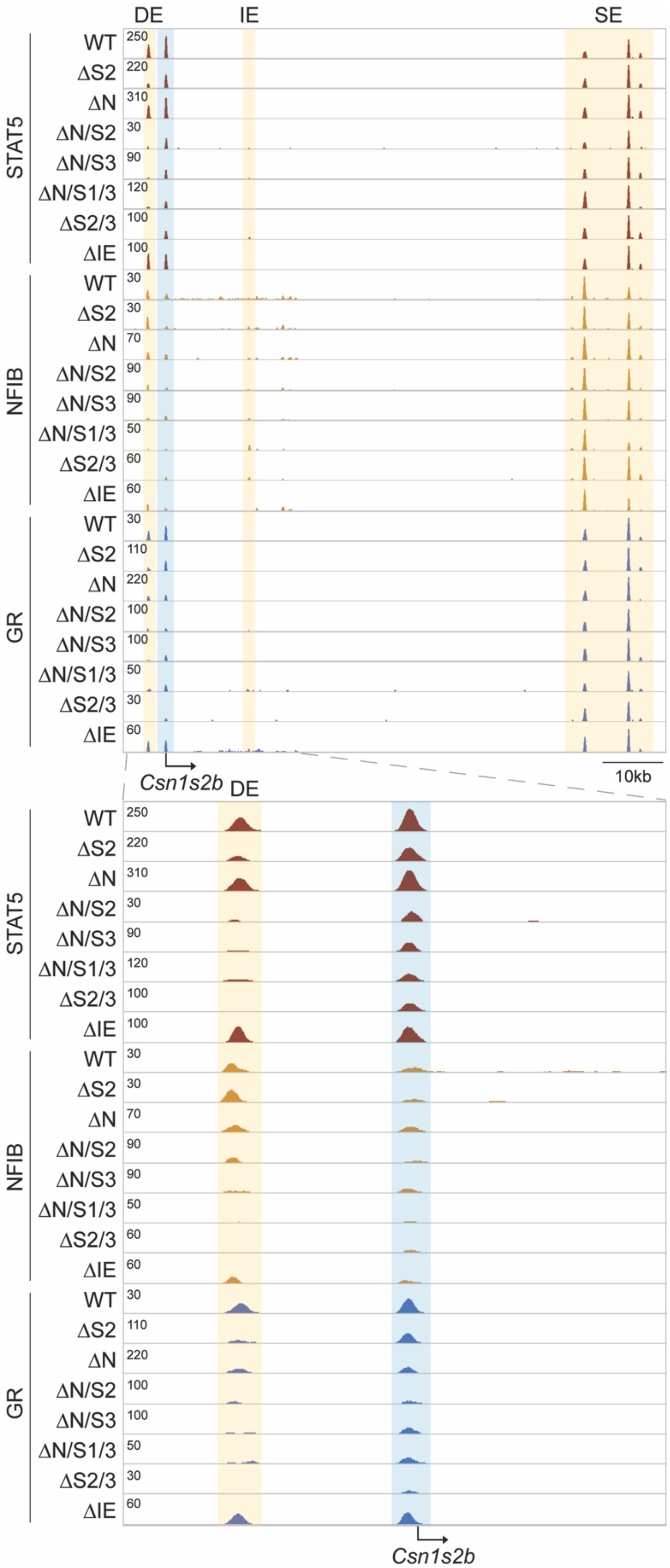

Supplementary Fig. 4 GR and STAT5 binding coincide. The SE served as ChIP-seq control for WT and mutant tissues at L10.

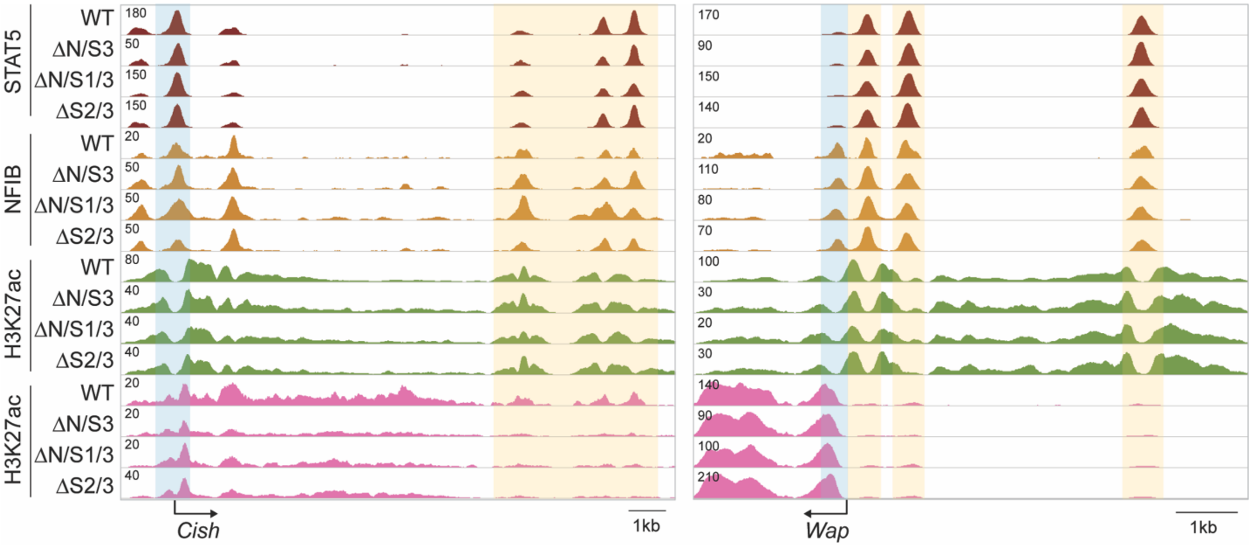

Supplementary Fig. 5 Critical role of the non-canonical STAT5 motif in the *Csn1s2b* distal enhancer. The *Cish* and *Wap* served as ChIP-seq control for WT and mutant tissues at day ten of lactation (L10).

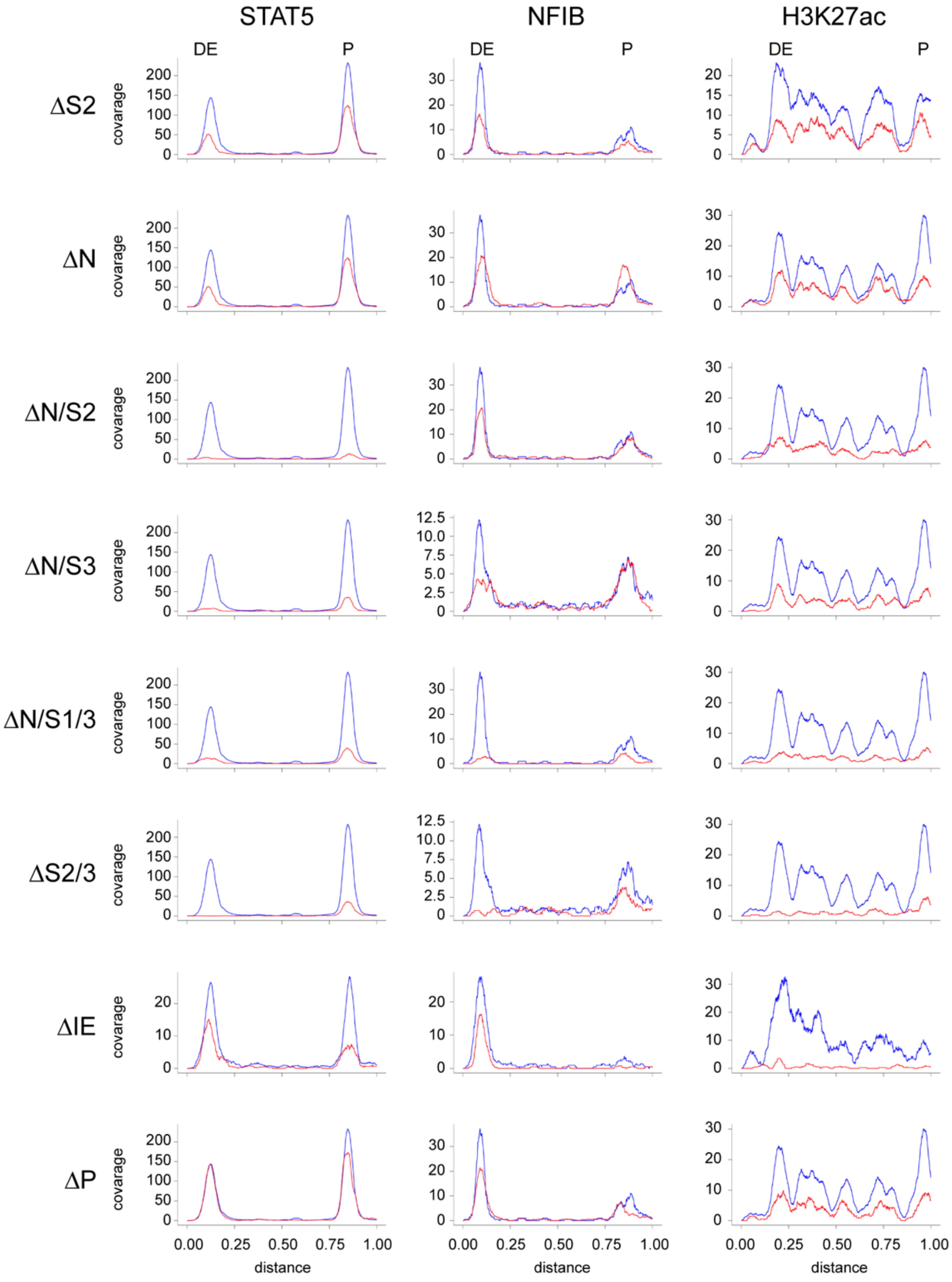

Supplementary Fig. 6 Genome-wide analyses of enhancer-promoter coverage in mammary tissue during lactation. Coverage plots of the *Csn1s2b* locus showing STAT5, NFIB, GR and H3K27ac in WT (blue) and mutant mice (red) at L10 and L1 (only for ΔIE).

Supplementary Table 1 Induction of *Csn* gene expression during pregnancy and lactation. mRNA levels of genes in the casein locus were measured by RNA-seq at day 6 of pregnancy (p6), day 1 of lactation (L1) and L10. The *Cish* gene serves control.

RNA-seq	Fold induction	
Gene	p6 > L1	L1 > L10
Csn1s1	44.67	10.7
Csn2	73.58	5.9
Csn3	57.14	7.8
Csn1s2a	104.05	5.7
Csn1s2b	358.27	253.8
Cish	1.26	1.5

Supplementary Table 2 Sequences of sgRNA of CRISPR/Cas9 targeted mice.

Target site	sgRNA sequences
NFIB motif	5’-AGAGATGGCAAGTGAGCTCA-3’
GAS motif	5’-ATGTTCTCTGAATCTATTCC-3’
Intronic enhancer	5’-AAACTGTGGTCTTCCAAGAA-3’
Promoter	5’-CAGGAATAAGTGATACAAATA-3’<br>5’-TCTGATATGGCAACTGTGTG-3’
SE	5’-CGCTAAGCATGTAGGGTCTC-3’<br>5’-TGGGTGTTCTTCCACTAGAC-3’

Supplementary Table 3 Characterization of mutant mice generated by CRISPR/Cas9. The GAS and NFIB binding motifs of the *Csn1s2b* enhancer are highlighted in yellow and blue, respectively. The GR half site is marked in underline.

Mice	Target sequence at Csn1s2b-DE
	N 126bp S1 S2
WT	TAAAGAGATGGCAAGTGAGCTCAGGGCTCT…ATGTTCTCTGAATCTATTCCTGGAAAAG
ΔS2 (1bp)	TAAAGAGATGGCAAGTGAGCTCAGGGCTCT…ATGTTCTCTGAATCTAT-CCTGGAAAAG
ΔN (15bp)	TAAAGAGATGGC---------------TCT…ATGTTCTCTGAATCTATTCCTGGAAAAG
ΔN/S2 (14/1bp)	TAAAGA--------------TCAGGGCTCT…ATGTTCTCTGAATCTAT-CCTGGAAAAG

Mice	Target sequence at Csn1s2b-DE
	N 104bp S3 18bp S1 S2
WT	AGATGGCAAGTGA…TTCTTCTGTTGAAATC…ATGTTCTCTGAATCTATTCCTGGAAAAG
ΔN/S3 (149bp)	AGATG--------…----------------…---TTCTCTGAATCTATTCCTGGAAAAG
ΔN/S1/3 (155bp)	AGATGGCCA----…----------------…------------TCTATTCCTGGAAAAG
ΔS2/3 (126bp)	AGATGGCAAGT--…----------------…ATGTTCTCTGAATCTAT-CCTGGAAAAG

Mice	Target sequence at Csn1s2b-IE
WT	TGGTCAAACTGTGGTCTTCCAAGAAAGGCATGAAGACTCTGCCAGATACCTTGGCCAAC
ΔIE-S (3bp)	TGGTCAAACTGTGGTCTTCCAA---AGGCATGAAGACTCTGCCAGATACCTTGGCCAAC
ΔIE-S (14bp)	TGGTCAAACTGTGG--------------CATGAAGACTCTGCCAGATACCTTGGCCAAC
ΔIE (36bp)	TGGTCAAA------------------------------------GATACCTTGGCCAAC

Mice	Target sequence at Csn1s2b-P
WT	TGACTTCTAAGAATACAAGTCCGTCTGAAGTTATTTTAAACCACACAGTTGCCATATCA
ΔP (18bp)	TGACTTCTAAGAATACAAGTCCGTCTGAAGTTA------------------CCATATCA

Mice	Target sequence at SE
	9kb
WT	AACCTCCTGAGACCCTACATGCTTAGCGTTT…GTGAGCTCACTCCTGTCTAGTGGAAGAACA
ΔSE	AACCTCCTGAG--------------------…--------------------TGGAAGAACA
