# Supplementary material for: Redundant and non-redundant cytokine-activated enhancers control Csn1s2b expression in the lactating mouse mammary gland
Source: Nat Commun. 2021 Apr 14;12:2239. doi: 10.1038/s41467-021-22500-w (PMC8047016; doi:10.1038/s41467-021-22500-w)
Supplement: Supplementary file 3 — Description of Additional Supplementary Files [file 41467_2021_22500_MOESM3_ESM.pdf]

Description of Additional Supplementary Files

File Name: Supplementary Data 1
Description: GEO numbers of ChIP-seq and RNA-seq data
