# Supplementary material for: Redundant and non-redundant cytokine-activated enhancers control Csn1s2b expression in the lactating mouse mammary gland
Source: Nat Commun. 2021 Apr 14;12:2239. doi: 10.1038/s41467-021-22500-w (PMC8047016; doi:10.1038/s41467-021-22500-w)
Supplement: Supplementary file 5 — Reporting Summary [file 41467_2021_22500_MOESM5_ESM.pdf]

nature research

Corresponding author(s): Lothar Hennighausen

Last updated by author(s): Feb 11, 2021

Reporting Summary

Nature Research wishes to improve the reproducibility of the work that we publish. This form provides structure for consistency and transparency in reporting. For further information on Nature Research policies, see our Editorial Policies and the Editorial Policy Checklist.

Statistics

For all statistical analyses, confirm that the following items are present in the figure legend, table legend, main text, or Methods section.

n/a	Confirmed	
☐	☒	The exact sample size (*n*) for each experimental group/condition, given as a discrete number and unit of measurement
☐	☒	A statement on whether measurements were taken from distinct samples or whether the same sample was measured repeatedly
☐	☒	The statistical test(s) used AND whether they are one- or two-sided<br>*Only common tests should be described solely by name; describe more complex techniques in the Methods section.*
☒	☐	A description of all covariates tested
☐	☒	A description of any assumptions or corrections, such as tests of normality and adjustment for multiple comparisons
☐	☒	A full description of the statistical parameters including central tendency (e.g. means) or other basic estimates (e.g. regression coefficient) AND variation (e.g. standard deviation) or associated estimates of uncertainty (e.g. confidence intervals)
☐	☒	For null hypothesis testing, the test statistic (e.g. *F*, *t*, *r*) with confidence intervals, effect sizes, degrees of freedom and *P* value noted<br>*Give P values as exact values whenever suitable.*
☒	☐	For Bayesian analysis, information on the choice of priors and Markov chain Monte Carlo settings
☒	☐	For hierarchical and complex designs, identification of the appropriate level for tests and full reporting of outcomes
☐	☒	Estimates of effect sizes (e.g. Cohen's *d*, Pearson's *r*), indicating how they were calculated

Our web collection on statistics for biologists contains articles on many of the points above.

Software and code

Policy information about availability of computer code

Data collection	ChIP-seq and RNA-seq data in GEO were downloaded using Sratoolkit (version 2.10.9) and newly generated ChIP-seq and RNA-seq reads were collected using HCS 3.4.0 software for HiSeq 3000 and HCS2.2.68 software for HiSeq 2500.
Data analysis	FastQC tool (version 0.11.9); Trimmomatic (version 0.36);Bowtie (version 1.2.2); Samtools (version 1.8); Picard (version 2.9.2); Homer (version 4.8.2); DeepTools (version 3.1.3); IGV(version 2.5.3); MACS (version 2.2.7.1); Bedtools (version 2.29.2); STAR RNA-seq (version 2.5.4a); HTSeq (version 0.9.1) ; R (version 3.6.3); Bioconductor (version 3.10); DESeq2; RUVSeq package; dplyr; ggplot2, GraphPad Prism 8 (version 8.2.0)

For manuscripts utilizing custom algorithms or software that are central to the research but not yet described in published literature, software must be made available to editors and reviewers. We strongly encourage code deposition in a community repository (e.g. GitHub). See the Nature Research guidelines for submitting code & software for further information.

Data

Policy information about availability of data

All manuscripts must include a data availability statement. This statement should provide the following information, where applicable:

- Accession codes, unique identifiers, or web links for publicly available datasets
- A list of figures that have associated raw data
- A description of any restrictions on data availability

All data were obtained or uploaded to Gene Expression Omnibus (GEO). ChIP-seq data of wild-type tissue at L1 and L10 were obtained under GSE74826, GSE115370, GSE145193, GSE127144 and GSE145193. RNA-seq data for WT at p18, L1 and L10 were downloaded from GSE127140 and GSE115370. The ChIP-seq and RNA-seq data from WT and mutant mice were uploaded in GSE161620. All files were summarized in Supplementary Table 4 and aligned to reference genome mm10.

Field-specific reporting

Please select the one below that is the best fit for your research. If you are not sure, read the appropriate sections before making your selection.

☒ Life sciences ☐ Behavioural & social sciences ☐ Ecological, evolutionary & environmental sciences

For a reference copy of the document with all sections, see nature.com/documents/nr-reporting-summary-flat.pdf

Life sciences study design

All studies must disclose on these points even when the disclosure is negative.

Sample size	11 mutant mouse lines with deletions of regulatory elements were generated. No statistical methods were used to determine sample size. In general, at least three independent replicates were performed in all experiments. When possible, we have aimed for the replication of the animal experiments in at least two different cohorts. The sample size used for each experiment is indicated at the corresponding figure legend in the manuscript.
Data exclusions	None of data were excluded in the data analysis.
Replication	The number of independent replicates for each experiment is indicated at the corresponding figure legend in the manuscript. In general, at least three independent replicates and two independent ChIP-seq replicates were performed.
Randomization	In all animal studies, groups were allocated randomly. Age and gender-matched animals were used in all the experiments.
Blinding	For all animal studies, the investigators were blind to group allocation. Blinding was not applicable to the rest of experiments.

Reporting for specific materials, systems and methods

We require information from authors about some types of materials, experimental systems and methods used in many studies. Here, indicate whether each material, system or method listed is relevant to your study. If you are not sure if a list item applies to your research, read the appropriate section before selecting a response.

Materials & experimental systems

n/a	Involved in the study	
☐	☒	Antibodies
☒	☐	Eukaryotic cell lines
☒	☐	Palaeontology and archaeology
☐	☒	Animals and other organisms
☒	☐	Human research participants
☒	☐	Clinical data
☒	☐	Dual use research of concern

Methods

n/a	Involved in the study	
☐	☒	ChIP-seq
☒	☐	Flow cytometry
☒	☐	MRI-based neuroimaging

Antibodies

Antibodies used	5-10 ug of antibodies were added in 1 mg of total proteins (1ml solution).<br>STAT5A (Santa Cruz Biotechnology, sc-1081 and sc-271542), GR (Thermo Fisher Scientific, PA1-511A), NFIB (Sigma-Aldrich, HPA003956), MED1 (Bethyl Laboratory, A300-793A), H3K27ac (Abcam, ab4729), RNA polymerase II (Abcam, ab5408), H3K4me1 (Active Motif, 39297) and H3K4me3 (Millipore, 07-473)
Validation	Previous papers from our group - PMID: 27376239, 30285185, 27694626, 32636391, 28009300, 28714474, 28334928, 32321991, 26446995, 27215382, 127139<br><br>STAT5A - PMID: 27376239, 30285185, 27694626, 32636391, 28009300, 28334928, 32321991, 26446995, 127139<br>GR - PMID: 27376239, 30285185, 27694626, 32636391, 28009300, 28334928, 127139<br>NFIB - PMID: 27376239<br>MED1 - PMID: 27376239, 30285185, 26446995<br>H3K27ac - PMID: 27376239, 30285185, 27694626, 32636391, 28009300, 28714474, 28334928, 32321991, 26446995, 127139<br>RNA polymerase II - PMID: 27376239, 30285185, 32636391, 28334928, 32321991, 26446995, 127139<br>H3K4me1 - PMID: 27215382, 127139<br>H3K4me3 - PMID: 27376239, 32636391, 28009300, 28714474, 32321991, 26446995, 27215382, 127139

Animals and other organisms

Policy information about studies involving animals; ARRIVE guidelines recommended for reporting animal research

Laboratory animals	C57BL/6N mice (Charles River Laboratories, MD) were used to generate CRISPR/Cas9 targeted mice and as a normal condition. All mice were 8-12 weeks and the mammary gland tissues at specific time points were harvested from pregnant or lactating females. Mice were housed in an environmentally controlled room (22-24 °C, with 50 ± 5% humidity and 12 h / 12 h light–dark cycle).
Wild animals	No wild animals were used in the study.
Field-collected samples	Mammary gland tissues from specific stages during pregnancy and lactation were harvested, and stored at -80°C.
Ethics oversight	All animals were housed and handled according to the guidelines of the Animal Care and Use Committee (ACUC) of the NIH (https://oacu.oir.nih.gov) and all animal experiments were approved by the ACUC of National Institute of Diabetes and Digestive and Kidney Diseases (NIDDK, MD) and performed under the NIDDK animal protocol K089-LGP-17.

Note that full information on the approval of the study protocol must also be provided in the manuscript.

ChIP-seq

Data deposition

☒ Confirm that both raw and final processed data have been deposited in a public database such as GEO.

☒ Confirm that you have deposited or provided access to graph files (e.g. BED files) for the called peaks.

Data access links
May remain private before publication.

https://www.ncbi.nlm.nih.gov/geo/query/acc.cgi?acc=GSE161620
secure token: klelgugifhgrdyf

Files in database submission

delCsn1s2b-DE-N_L10_GR_rep1.fastq.gz
delCsn1s2b-DE-N_L10_GR_rep2.fastq.gz
delCsn1s2b-DE-N_L10_H3K27ac_rep1.fastq.gz
delCsn1s2b-DE-N_L10_H3K27ac_rep2.fastq.gz
delCsn1s2b-DE-N_L10_NFIB_rep1.fastq.gz
delCsn1s2b-DE-N_L10_NFIB_rep2.fastq.gz
delCsn1s2b-DE-N_L10_PolII_rep1.fastq.gz
delCsn1s2b-DE-N_L10_PolII_rep2.fastq.gz
delCsn1s2b-DE-N_L10_STAT5_rep1.fastq.gz
delCsn1s2b-DE-N_L10_STAT5_rep2.fastq.gz
delCsn1s2b-DE-N-S1-S3_L10_GR_rep1.fastq.gz
delCsn1s2b-DE-N-S1-S3_L10_GR_rep2.fastq.gz
delCsn1s2b-DE-N-S1-S3_L10_H3K27ac_rep1.fastq.gz
delCsn1s2b-DE-N-S1-S3_L10_H3K27ac_rep2.fastq.gz
delCsn1s2b-DE-N-S1-S3_L10_NFIB_rep1.fastq.gz
delCsn1s2b-DE-N-S1-S3_L10_NFIB_rep2.fastq.gz
delCsn1s2b-DE-N-S1-S3_L10_PolII_rep1.fastq.gz
delCsn1s2b-DE-N-S1-S3_L10_PolII_rep2.fastq.gz
delCsn1s2b-DE-N-S1-S3_L10_STAT5_rep1.fastq.gz
delCsn1s2b-DE-N-S1-S3_L10_STAT5_rep2.fastq.gz
delCsn1s2b-DE-N-S2_L10_GR_rep1.fastq.gz
delCsn1s2b-DE-N-S2_L10_GR_rep2.fastq.gz
delCsn1s2b-DE-N-S2_L10_H3K27ac_rep1.fastq.gz
delCsn1s2b-DE-N-S2_L10_H3K27ac_rep2.fastq.gz
delCsn1s2b-DE-N-S2_L10_NFIB_rep1.fastq.gz
delCsn1s2b-DE-N-S2_L10_NFIB_rep2.fastq.gz
delCsn1s2b-DE-N-S2_L10_PolII_rep1.fastq.gz
delCsn1s2b-DE-N-S2_L10_PolII_rep2.fastq.gz
delCsn1s2b-DE-N-S2_L10_STAT5_rep1.fastq.gz
delCsn1s2b-DE-N-S2_L10_STAT5_rep2.fastq.gz
delCsn1s2b-DE-N-S3_L10_GR_rep1.fastq.gz
delCsn1s2b-DE-N-S3_L10_GR_rep2.fastq.gz
delCsn1s2b-DE-N-S3_L10_H3K27ac_rep1.fastq.gz
delCsn1s2b-DE-N-S3_L10_H3K27ac_rep2.fastq.gz
delCsn1s2b-DE-N-S3_L10_NFIB_rep1.fastq.gz
delCsn1s2b-DE-N-S3_L10_NFIB_rep2.fastq.gz
delCsn1s2b-DE-N-S3_L10_PolII_rep1.fastq.gz
delCsn1s2b-DE-N-S3_L10_PolII_rep2.fastq.gz
delCsn1s2b-DE-N-S3_L10_STAT5_rep1.fastq.gz
delCsn1s2b-DE-N-S3_L10_STAT5_rep2.fastq.gz
delCsn1s2b-DE-S2_L10_GR_rep1.fastq.gz
delCsn1s2b-DE-S2_L10_GR_rep2.fastq.gz
delCsn1s2b-DE-S2_L10_H3K27ac_rep1.fastq.gz
delCsn1s2b-DE-S2_L10_H3K27ac_rep2.fastq.gz
delCsn1s2b-DE-S2_L10_NFIB_rep1.fastq.gz

delCsn1s2b-DE-S2_L10_NFIB_rep2.fastq.gz
delCsn1s2b-DE-S2_L10_PolII_rep1.fastq.gz
delCsn1s2b-DE-S2_L10_PolII_rep2.fastq.gz
delCsn1s2b-DE-S2_L10_STAT5_rep1.fastq.gz
delCsn1s2b-DE-S2_L10_STAT5_rep2.fastq.gz
delCsn1s2b-DE-S2-S3_L10_GR_rep1.fastq.gz
delCsn1s2b-DE-S2-S3_L10_GR_rep2.fastq.gz
delCsn1s2b-DE-S2-S3_L10_H3K27ac_rep1.fastq.gz
delCsn1s2b-DE-S2-S3_L10_H3K27ac_rep2.fastq.gz
delCsn1s2b-DE-S2-S3_L10_NFIB_rep1.fastq.gz
delCsn1s2b-DE-S2-S3_L10_NFIB_rep2.fastq.gz
delCsn1s2b-DE-S2-S3_L10_PolII_rep1.fastq.gz
delCsn1s2b-DE-S2-S3_L10_PolII_rep2.fastq.gz
delCsn1s2b-DE-S2-S3_L10_STAT5_rep1.fastq.gz
delCsn1s2b-DE-S2-S3_L10_STAT5_rep2.fastq.gz
delCsn1s2b-IE_L1_H3K27ac_rep1.fastq.gz
delCsn1s2b-IE_L1_NFIB_rep1.fastq.gz
delCsn1s2b-IE_L1_PolII_rep1.fastq.gz
delCsn1s2b-IE_L1_STAT5_rep1.fastq.gz
delCsn1s2b-IE_L1_STAT5_rep2.fastq.gz
delCsn1s2b-IE_L10_GR_rep1.fastq.gz
delCsn1s2b-IE_L10_GR_rep2.fastq.gz
delCsn1s2b-IE_L10_H3K27ac_rep1.fastq.gz
delCsn1s2b-IE_L10_H3K27ac_rep2.fastq.gz
delCsn1s2b-IE_L10_NFIB_rep1.fastq.gz
delCsn1s2b-IE_L10_NFIB_rep2.fastq.gz
delCsn1s2b-IE_L10_PolII_rep1.fastq.gz
delCsn1s2b-IE_L10_PolII_rep2.fastq.gz
delCsn1s2b-IE_L10_STAT5_rep1.fastq.gz
delCsn1s2b-IE_L10_STAT5_rep2.fastq.gz
delCsn1s2b-P_L10_GR_rep1.fastq.gz
delCsn1s2b-P_L10_H3K4me1_rep1.fastq.gz
delCsn1s2b-P_L10_H3K4me3_rep1.fastq.gz
delCsn1s2b-P_L10_H3K27ac_rep1.fastq.gz
delCsn1s2b-P_L10_NFIB_rep1.fastq.gz
delCsn1s2b-P_L10_PolII_rep1.fastq.gz
delCsn1s2b-P_L10_STAT5_rep1.fastq.gz
delS1-4_L1_NFIB_rep1.fastq.gz
delS1-4_L1_NFIB_rep2.fastq.gz
WT_L1_PolII_rep1.fastq.gz
WT_L1_PolII_rep2.fastq.gz
WT_L10_H3K4me1_rep1.fastq.gz
WT_L10_H3K4me1_rep2.fastq.gz
WT_L10_H3K4me1_rep3.fastq.gz
WT_L10_H3K4me3_rep1.fastq.gz
WT_L10_H3K4me3_rep2.fastq.gz
WT_L10_NFIB_rep1.fastq.gz
WT_L10_NFIB_rep2.fastq.gz
WT_p18_GR_rep1.fastq.gz
WT_p18_GR_rep2.fastq.gz
WT_p18_H3K27ac_rep1.fastq.gz
WT_p18_H3K27ac_rep2.fastq.gz
WT_p18_PolII_rep1.fastq.gz
WT_p18_STAT5_rep1.fastq.gz
WT_p18_STAT5_rep2.fastq.gz
delCsn1s2b-DE-N_L10_GR_rep1.ucsc.bedGraph.gz.tdf
delCsn1s2b-DE-N_L10_GR_rep2.ucsc.bedGraph.gz.tdf
delCsn1s2b-DE-N_L10_H3K27ac_rep1.ucsc.bedGraph.gz.tdf
delCsn1s2b-DE-N_L10_H3K27ac_rep2.ucsc.bedGraph.gz.tdf
delCsn1s2b-DE-N_L10_NFIB_rep1.ucsc.bedGraph.gz.tdf
delCsn1s2b-DE-N_L10_NFIB_rep2.ucsc.bedGraph.gz.tdf
delCsn1s2b-DE-N_L10_PolII_rep1.ucsc.bedGraph.gz.tdf
delCsn1s2b-DE-N_L10_PolII_rep2.ucsc.bedGraph.gz.tdf
delCsn1s2b-DE-N_L10_STAT5_rep1.ucsc.bedGraph.gz.tdf
delCsn1s2b-DE-N_L10_STAT5_rep2.ucsc.bedGraph.gz.tdf
delCsn1s2b-DE-N-S1-S3_L10_GR_rep1.ucsc.bedGraph.gz.tdf
delCsn1s2b-DE-N-S1-S3_L10_GR_rep2.ucsc.bedGraph.gz.tdf
delCsn1s2b-DE-N-S1-S3_L10_H3K27ac_rep1.ucsc.bedGraph.gz.tdf
delCsn1s2b-DE-N-S1-S3_L10_H3K27ac_rep2.ucsc.bedGraph.gz.tdf
delCsn1s2b-DE-N-S1-S3_L10_NFIB_rep1.ucsc.bedGraph.gz.tdf
delCsn1s2b-DE-N-S1-S3_L10_NFIB_rep2.ucsc.bedGraph.gz.tdf
delCsn1s2b-DE-N-S1-S3_L10_PolII_rep1.ucsc.bedGraph.gz.tdf
delCsn1s2b-DE-N-S1-S3_L10_PolII_rep2.ucsc.bedGraph.gz.tdf
delCsn1s2b-DE-N-S1-S3_L10_STAT5_rep1.ucsc.bedGraph.gz.tdf
delCsn1s2b-DE-N-S1-S3_L10_STAT5_rep2.ucsc.bedGraph.gz.tdf
delCsn1s2b-DE-N-S2_L10_GR_rep1.ucsc.bedGraph.gz.tdf

delCsn1s2b-DE-N-S2_L10_GR_rep2.ucsc.bedGraph.gz.tdf
delCsn1s2b-DE-N-S2_L10_H3K27ac_rep1.ucsc.bedGraph.gz.tdf
delCsn1s2b-DE-N-S2_L10_H3K27ac_rep2.ucsc.bedGraph.gz.tdf
delCsn1s2b-DE-N-S2_L10_NFIB_rep1.ucsc.bedGraph.gz.tdf
delCsn1s2b-DE-N-S2_L10_NFIB_rep2.ucsc.bedGraph.gz.tdf
delCsn1s2b-DE-N-S2_L10_PolII_rep1.ucsc.bedGraph.gz.tdf
delCsn1s2b-DE-N-S2_L10_PolII_rep2.ucsc.bedGraph.gz.tdf
delCsn1s2b-DE-N-S2_L10_STAT5_rep1.ucsc.bedGraph.gz.tdf
delCsn1s2b-DE-N-S2_L10_STAT5_rep2.ucsc.bedGraph.gz.tdf
delCsn1s2b-DE-N-S3_L10_GR_rep1.ucsc.bedGraph.gz.tdf
delCsn1s2b-DE-N-S3_L10_GR_rep2.ucsc.bedGraph.gz.tdf
delCsn1s2b-DE-N-S3_L10_H3K27ac_rep1.ucsc.bedGraph.gz.tdf
delCsn1s2b-DE-N-S3_L10_H3K27ac_rep2.ucsc.bedGraph.gz.tdf
delCsn1s2b-DE-N-S3_L10_NFIB_rep1.ucsc.bedGraph.gz.tdf
delCsn1s2b-DE-N-S3_L10_NFIB_rep2.ucsc.bedGraph.gz.tdf
delCsn1s2b-DE-N-S3_L10_PolII_rep1.ucsc.bedGraph.gz.tdf
delCsn1s2b-DE-N-S3_L10_PolII_rep2.ucsc.bedGraph.gz.tdf
delCsn1s2b-DE-N-S3_L10_STAT5_rep1.ucsc.bedGraph.gz.tdf
delCsn1s2b-DE-N-S3_L10_STAT5_rep2.ucsc.bedGraph.gz.tdf
delCsn1s2b-DE-S2_L10_GR_rep1.ucsc.bedGraph.gz.tdf
delCsn1s2b-DE-S2_L10_GR_rep2.ucsc.bedGraph.gz.tdf
delCsn1s2b-DE-S2_L10_H3K27ac_rep1.ucsc.bedGraph.gz.tdf
delCsn1s2b-DE-S2_L10_H3K27ac_rep2.ucsc.bedGraph.gz.tdf
delCsn1s2b-DE-S2_L10_NFIB_rep1.ucsc.bedGraph.gz.tdf
delCsn1s2b-DE-S2_L10_NFIB_rep2.ucsc.bedGraph.gz.tdf
delCsn1s2b-DE-S2_L10_PolII_rep1.ucsc.bedGraph.gz.tdf
delCsn1s2b-DE-S2_L10_PolII_rep2.ucsc.bedGraph.gz.tdf
delCsn1s2b-DE-S2_L10_STAT5_rep1.ucsc.bedGraph.gz.tdf
delCsn1s2b-DE-S2_L10_STAT5_rep2.ucsc.bedGraph.gz.tdf
delCsn1s2b-DE-S2-S3_L10_GR_rep1.ucsc.bedGraph.gz.tdf
delCsn1s2b-DE-S2-S3_L10_GR_rep2.ucsc.bedGraph.gz.tdf
delCsn1s2b-DE-S2-S3_L10_H3K27ac_rep1.ucsc.bedGraph.gz.tdf
delCsn1s2b-DE-S2-S3_L10_H3K27ac_rep2.ucsc.bedGraph.gz.tdf
delCsn1s2b-DE-S2-S3_L10_NFIB_rep1.ucsc.bedGraph.gz.tdf
delCsn1s2b-DE-S2-S3_L10_NFIB_rep2.ucsc.bedGraph.gz.tdf
delCsn1s2b-DE-S2-S3_L10_PolII_rep1.ucsc.bedGraph.gz.tdf
delCsn1s2b-DE-S2-S3_L10_PolII_rep2.ucsc.bedGraph.gz.tdf
delCsn1s2b-DE-S2-S3_L10_STAT5_rep1.ucsc.bedGraph.gz.tdf
delCsn1s2b-DE-S2-S3_L10_STAT5_rep2.ucsc.bedGraph.gz.tdf
delCsn1s2b-IE_L1_H3K27ac_rep1.ucsc.bedGraph.gz.tdf
delCsn1s2b-IE_L1_NFIB_rep1.ucsc.bedGraph.gz.tdf
delCsn1s2b-IE_L1_PolII_rep1.ucsc.bedGraph.gz.tdf
delCsn1s2b-IE_L1_STAT5_rep1.ucsc.bedGraph.gz.tdf
delCsn1s2b-IE_L1_STAT5_rep2.ucsc.bedGraph.gz.tdf
delCsn1s2b-IE_L10_GR_rep1.ucsc.bedGraph.gz.tdf
delCsn1s2b-IE_L10_GR_rep2.ucsc.bedGraph.gz.tdf
delCsn1s2b-IE_L10_H3K27ac_rep1.ucsc.bedGraph.gz.tdf
delCsn1s2b-IE_L10_H3K27ac_rep2.ucsc.bedGraph.gz.tdf
delCsn1s2b-IE_L10_NFIB_rep1.ucsc.bedGraph.gz.tdf
delCsn1s2b-IE_L10_NFIB_rep2.ucsc.bedGraph.gz.tdf
delCsn1s2b-IE_L10_PolII_rep1.ucsc.bedGraph.gz.tdf
delCsn1s2b-IE_L10_PolII_rep2.ucsc.bedGraph.gz.tdf
delCsn1s2b-IE_L10_STAT5_rep1.ucsc.bedGraph.gz.tdf
delCsn1s2b-IE_L10_STAT5_rep2.ucsc.bedGraph.gz.tdf
delCsn1s2b-P_L10_GR_rep1.ucsc.bedGraph.gz.tdf
delCsn1s2b-P_L10_H3K4me1_rep1.ucsc.bedGraph.gz.tdf
delCsn1s2b-P_L10_H3K4me3_rep1.ucsc.bedGraph.gz.tdf
delCsn1s2b-P_L10_H3K27ac_rep1.ucsc.bedGraph.gz.tdf
delCsn1s2b-P_L10_NFIB_rep1.ucsc.bedGraph.gz.tdf
delCsn1s2b-P_L10_PolII_rep1.ucsc.bedGraph.gz.tdf
delCsn1s2b-P_L10_STAT5_rep1.ucsc.bedGraph.gz.tdf
delS1-4_L1_NFIB_rep1.ucsc.bedGraph.gz.tdf
delS1-4_L1_NFIB_rep2.ucsc.bedGraph.gz.tdf
WT_L1_PolII_rep1.ucsc.bedGraph.gz.tdf
WT_L1_PolII_rep2.ucsc.bedGraph.gz.tdf
WT_L10_H3K4me1_rep1.ucsc.bedGraph.gz.tdf
WT_L10_H3K4me1_rep2.ucsc.bedGraph.gz.tdf
WT_L10_H3K4me1_rep3.ucsc.bedGraph.gz.tdf
WT_L10_H3K4me3_rep1.ucsc.bedGraph.gz.tdf
WT_L10_H3K4me3_rep2.ucsc.bedGraph.gz.tdf
WT_L10_NFIB_rep1.ucsc.bedGraph.gz.tdf
WT_L10_NFIB_rep2.ucsc.bedGraph.gz.tdf
WT_p18_GR_rep1.ucsc.bedGraph.gz.tdf
WT_p18_GR_rep2.ucsc.bedGraph.gz.tdf
WT_p18_H3K27ac_rep1.ucsc.bedGraph.gz.tdf
WT_p18_H3K27ac_rep2.ucsc.bedGraph.gz.tdf

	WT_p18_PolII_rep1.ucsc.bedGraph.gz.tdf<br>WT_p18_STAT5_rep1.ucsc.bedGraph.gz.tdf<br>WT_p18_STAT5_rep2.ucsc.bedGraph.gz.tdf
Genome browser session (e.g. UCSC)	https://genome.ucsc.edu/cgi-bin/hgTracks?db=mm10&lastVirtModeType=default&lastVirtModeExtraState=&virtModeType=default&virtMode=0&nonVirtPosition=&position=chr5%3A87762015%2D87894679&hgsid=957092287_8igAv0Gisx9hbeD2tnkjcrRGPtAg

Methodology

Replicates	WT-p18-PolII, Csn1s2b-IE-L1 and Csn1s2b-P-L10 samples have one data set because they were not critical for the study. For all other ChIP-seq experiments more than two replicates were conducted.
Sequencing depth	All Sequencing was done as 51bp single end sequence. Sequencing was done to achieve > 30 million reads per biological replicate.
Antibodies	STAT5A (Santa Cruz Biotechnology, sc-1081 and sc-271542), GR (Thermo Fisher Scientific, PA1-511A), NFIB (Sigma-Aldrich, HPA003956), MED1 (Bethyl Laboratory, A300-793A), H3K27ac (Abcam, ab4729), RNA polymerase II (Abcam, ab5408), H3K4me1 (Active Motif, 39297) and H3K4me3 (Millipore, 07-473)
Peak calling parameters	MACS2 was used with default settings for peak calling.
Data quality	> 20000 peaks for transcription factors and > 100000 peaks for histone markers by q-value (< 0.001 for TFs, 0.1 or 0.5 for histone markers) were at 5% FDR and above 4-fold enrichment.
Software	FastQC tool (version 0.11.9); Trimmomatic (version 0.36);Bowtie (version 1.2.2); Samtools (version 1.8); Picard; Homer (version 4.8.2); DeepTools (version 3.1.3); IGV(version 2.5.3); MACS (version 2.2.7.1); Bedtools (version 2.29.2)
